# Reviews and Book Notices

**Published:** 1878-08

**Authors:** 


### Reviews and Book Notices.

HOUSE DRAINAGE AND WATER SERVICE IN CITIES, VILLAGES AND RURAL NEIGHBORHOODS, WITH INCIDENTAL CONSIDERATION OF CAUSES AFFECTING THE HEALTHFULNESS OF DWELLINGS. By James C. Bayles, editor of *Iron Age* and *The Metal Worker*. Pp. 351, cloth. Published by David Williams, 83 Reade St., New York, 1878.

This work is the practical development of an extensive correspondence and discussion in *The Metal Worker*, of questions pertaining to sanitary engineering. It was not, however, until the author became aware of the necessity of such a work by the appreciation on the part of plumbers of his two papers read before the Public Health Association of New York, on house drainage, that he resolved on publication. Were our words likely to reach the plumbers, we should recommend this work to them in terms of the highest commendation. But naturally it is more with reference to the relation which it bears to the profession, that we consider the volume.

The object which the author sets before him is well expressed by the title of the book, and whether we refer to the chapter on sewer gas, waste and soil pipes, traps and seals and the ventilation of soil pipes, the chemistry of plumbing, or sanitary construction and drainage of country houses, we find that the author is at once comprehensive, lucid, concise and eminently practical. He justly deprecates the niggardliness which is too frequently displayed, even by men practical on other points, when the question of plumbing is the subject of consideration; and shows the results of such supposed economy in the frequent repairs necessitated in the actual plumbing, and the unavoidable and often incomprehensible continuation of ill-health and untimely death. To show the importance of the work which Mr. B. has undertaken, we need only refer the reader to the very important report of the Officer of Health for Glasgow, which he read before the Philosophical Society of Glasgow. This paper appears in full in the *British Medical Journal* for May 11, 1878, and shows unmistakably the relation between the waste-pipe service of the houses and the prevalence of diphtheria and enteric fever. We only regret, since reading Mr. B.'s book, that the construction of the traps in the Health Officer's report were not more definitely recorded.

Mr. Bayles' remarks on the action of the different kinds of water on lead cannot fail to be interesting to practitioners ; it might even be said that those who remain ignorant of such facts are positively culpable.

The following extract gives Mr. Bayles' own experience in the matter of earth-closets (p. 272) :

"From personal experience, and after the severest test which I could devise, I can recommend the earth-closet as the best, cheapest and most generally satisfactory of indoor commodes for country houses.

"There are several forms of earth-closets in the market. From $20 to $25 is the price of one made after the most approved pattern, with a capacious hopper, and an arrangement for discharging a fixed quantity of earth into the receiver. Those who are able and willing to pay this price will get a good article, with full directions for its use and care. For the benefit of those who are not, it may be said that a convenient earth-closet can easily be made at a small expense, and without infringing anybody's patents, by any person with intelligence enough to build a hen-coop. The writer's experience in building and managing an earth-closet may not be without interest. It was made of pine boards in the shape shown in Fig. 26, being simply a box with two covers and no bottom. The under cover, which served as the seat, was hinged to the edge of the box, and the upper cover was hinged to the lower, so that they could be easily raised, singly or together, as desired, without interfering with each other. Under the seat, and standing upon the floor, was placed a galvanized iron coal-hod. A tin pail full of dry, sifted earth stood beside it. When two or three inches of earth had been sprinkled upon the bottom of the coal-hod, the earth-closet was ready for use. The whole cost of the apparatus, including a large coal-hod, did not exceed $3.50, but it was as satisfactory as one could be. A small shovelful of earth was thrown in when the closet was used, and it was perfectly free from unpleasant odor, though it was daily used by several persons. The only attention it needed or received was to empty the hod when full.

"A somewhat more convenient shape for the box would have been to make it long enough to admit of partitioning off one end for an earth reservoir, as shown in Fig. 27. This would dispense with the pail for holding the earth, and render the whole apparatus complete in itself." R. T.

Defects of Vision. By Robert Bradwell Carter, F. R. C. S. McMillan and Company, London, publishers, 1877.

This is another happy effort of the author of a work on Diseases of the Eye, published in 1875. It is in the form of lectures, six in number, delivered before the Royal College of Surgeons of England, and is notable for its directness of purpose, the brief, yet thorough manner in which this subject is treated, and for the practical value of its contents.

The first two lectures definitely mark "the chief features of the three great factors of sustained vision—refraction, accommodation and convergence."

The dioptric system, the metric system in its relation to lenses, is explained, showing an advantage for it over the old system of inches, in the absence of fractions in necessary calculations, and in the greater regularity of the intervals between the series of lenses. It offers, too, a wider range of lenses, admitting of any useful combination. The author suggests that in prescribing glasses fitted by the French trial lenses, marked in inches, the prescription is for glasses in French inches, whereas the optician fills it by English measurement, thus giving rise to mistakes easily prevented by the new system.

He calls special attention to the difference between the terms *range* and *region* of accommodation; *range* meaning the *power*

of accommodation; *region*, "the limits of space within which the *range* is exercised."

The remaining four lectures are given to the consideration of presbyopia, myopia and hypermetropia, astigmatism, and asthenopia, the principal forms of "conditioned vision."

The subject of astigmatism is treated intelligibly, and is made very simple. All questions which perplex instead of assisting us are excluded. About one page of the volume is devoted to a thorough description of all the varieties of astigmatism, with the figures illustrating the subject. *Multum in parvo* seems to be the ruling principle.

The author is fair in giving to American ophthalmologists credit for what they have done, a rare thing in European writers.

The book is interesting, and one who reads it finishes with two feelings: the *first* is one of satisfaction that so much available knowledge is to be found in so small a compass; the *second* is a regret that more of the books written are not like it in brevity and excellence.

It is an octavo volume, which the publishers have sent out printed in clear, large type, on tinted paper, and neatly bound.

S. O. R.

SYNOPSIS OF THE DISEASES OF THE LARYNX, LUNGS AND HEART, COMPRISING DR. EDWARDS' TABLES ON THE EXAMINATION OF THE CHEST, WITH ALTERATIONS AND ADDITIONS. By F. DeHavilland Hall, M. D., London. J. A. Churchill, London, 1878.

This little book contains in a very concise form, and arranged in a manner easily available for the student, the information which usually fills a large volume. It is a chart for the student and practitioner, which will at once refresh their memories and point out thoracic disease in an unmistakable manner. In it the regions of the chest with their contents, and the physical signs of health and disease are so tabulated that their value may be appreciated at a glance; and the symptoms and signs of various diseases are compared, so as to make diagnosis easy.

A PRACTICAL MANUAL OF THE DISEASES OF CHILDREN, WITH A FORMULARY. By Edward Ellis, M. D., late Senior Physician to the Victoria Hospital for Sick Children, etc., etc. Third edition; pp. 390. Lindsay & Blackiston, 1878.

A very excellent little work, characterized by short yet faithful descriptions of disease. A book not large enough to contain all which might be said in regard to diseases of children, or indeed to treat any one disease in an exhaustive manner, but rather a work in which the great majority of children's complaints are truly and vividly portrayed with a practical, and, in the main, recent treatment. The author's aim, as expressed in his preface to the first edition, is certainly realized. It is "concise" and a "handy book of reference." The writer of this review has carefully read the book and will faithfully maintain his proposition with regard to its exceeding usefulness. A few, from the many very excellent sections will be briefly noticed, and with the earnest desire to aid the author in producing a perfect book, if another edition should ever be needed, we will venture a few suggestions.

The chapter on general management and diet is remarkably good and contains some suggestions which we have not noticed in other and larger works. In the list of foods for infants, however, we do not notice oatmeal; a fact which excites some surprise, for it certainly has an established reputation in babies' dietary.

The section on apnœa is hardly as complete as one could wish, the sthenic and asthenic varieties not being sufficiently differentiated to guide an inexperienced practitioner. In dressing the navel we are advised, as of old, "to wrap it in a piece of soft linen in which a hole has been made for it to come through." A more elegant procedure, and one equally safe, is to follow the directions of Prof. Goodell, of Philadelphia.

The description of malarial diseases in this work, and indeed in most books on diseases of children, will not suffice for this climate. We not only have an *intermittent*, but we have a *remittent* form of fever due to malaria, which is neither typhoid or associated with the typhoid condition. And we, perhaps, may say here, as well as at at any other place, that remittent fever in children is the disease above all others which we are liable to confound with the first stage of tubercular meningitis. The author of this work speaks only of typhoid fever as a disease liable to be confounded with this terribly fatal meningeal inflammation.

The distinction between croup and diphtheria is very fully given, and, while recognizing the high authority of those who urge their identity, our author remains unconvinced. He confesses a family likeness between not only diphtheria and croup, but scarlatinous sore throat with patches of exudation. The asthenia, epidemy, and marked contagiousness of the first, compared with the sthenic, sporadic, and doubtfully contagious character of the second, are believed to mark two maladies. The use of the bichromate of potash is highly spoken of in diphtheria. The section on diseases of the heart is excellent. The chapter devoted to diseases of the food passages and abdominal organs. contains much which is valuable, although we must be allowed to differ with the author's classification and express surprise that so much space, comparatively, is given to gastritis, a disease of very rare occurrence, and such brief mention made of those very frequent and formidable diseases of children, indigestion and entero-colitis. The number of American vegetable remedies suggested and recommended is remarkable.

A few errors remain even in this third edition. The phrase "weak Condy's fluid lotion" appears two or three times; the word *when* is omitted at line 22 p. 287, and finally Dr. Eustace Smith is made to describe the "tongue of worms." C. W. E.

PRACTICAL CHEMISTRY FOR MEDICAL STUDENTS. Specially arranged for the First M. B. Course. By M. M. Pattison Muir, F. R. S. E., Prælector in Chemistry, Gouville and Gains College, Cambridge. London: Macmillan & Co. Chicago, Jansen, McClurg & Co. 1878. Cloth, pp. 64. 60c.

This is a little laboratory aid, and as such will be found very useful. We think it might have been made more serviceable by the introduction of formulæ. The bases are arranged in five groups, and the acids into six. The appendix contains the application of the various tests in a tabulated form, together with the tests for a few of the alkaloids. R. P.

